# Nowruz travelers and the COVID-19 pandemic in Iran

**DOI:** 10.1017/ice.2020.152

**Published:** 2020-04-20

**Authors:** Amir Kaffashi, Frieda Jahani

**Affiliations:** Razi Vaccine and Serum Research Institute, Agricultural Research, Education and Extension Organization (AREEO), Karaj, Iran

*To the Editor*—Iran, struggling with the toughest sanctions, is facing serious challenges in containing the pandemic of coronavirus disease 2019 (COVID-19). As of April 9, 2020, some 66,220 confirmed cases and 4,110 deaths had been reported in Iran.^1^ The rise of this pandemic coincided with Nowruz (the Persian New Year) holidays, which started on March 19, 2020, and lasted ~2 weeks. Millions of Iranians travel around the country during the Nowruz holiday.

Iranian authorities urged people to stay home during Nowruz and announced that public screening to detect coronavirus would be underway at airports, railway stations, bus terminals, and the city entrances and exits.^2^ By March 22, 2020, however, ~8,700,000 people in transit and the passengers of ~2,900,000 cars had been screened using thermal imager and gun thermometers. Among them, 6,464 were referred to clinical centers for further examination.^3^ Given the fact people with no symptoms might have been missed during public screening, a large number of travelers spread the SARS-CoV-2 virus across the country. The implications of virus transmission from persons with asymptomatic or very mild symptomatic cases of COVID-19 has already been considered vital for the formulation of containment strategies.^4^

In a similar situation, the COVID-19 epidemic in China coincided with the peak of the Chinese spring festival holidays. The Chinese government implemented rigorous quarantine measures and locked down the epicenter of the outbreak, Wuhan City, Hubei Province, on January 23, 2020. No new confirmed cases occurred in most provinces across China and in most cities in Hubei Province for >10 consecutive days by March 1, 2020, suggesting that the lockdown had been effective in mitigating the spread of the virus.^5^ Apart from social and economic effects, such travel restrictions are considered less effective once the outbreak is more widespread.^6^ By March 9, 2020, COVID-19 cases had been reported in all provinces in Iran, at incidence rates ranging from 0.8 cases per 100,000 population in Bushehr to 61.8 cases per 100,000 population in Qom.^7^ It was already too late for any restriction to be effective.

On March 27, 2020, the Iranian government implemented a travel ban preventing people from leaving their cities and requesting those already on trips to return at their earliest opportunity.^8^ However, a modeling study showed that travel limitations eliminating up to 90% of the traffic have a modest effect and that such measures should be combined with public health interventions and behavioral changes to achieve a considerable reduction in disease transmission.^9^ The spread of SARS-CoV-2 is not yet slowing down in Iran, and the recommendation of the World Health Organization (WHO) seems more likely to result in the containment of COVID-19. These include widespread testing, quarantine of cases, contact tracing, and social distancing, which were successfully implemented by South Korea.^10^
